# Swine-plague in Austria

**Published:** 1896-04

**Authors:** 


### ABSTRACTS FROM FOREIGN JOURNALS.

THE SWINE-PLAGUE (SWINEPEST) IN AUSTRIA. The following abstract is taken from the report of Prof. Hugo Schindelka, member of a committee appointed by the Veterinary Association of lower Austria to collect statistics and report upon the disease, printed in the *Thierärztliches Centralblatt* of January 1 and 15, February 1 and 15, 1896.

As is now pretty well known, we were surprised in the early months of 1895 by the breaking out of an epidemic among swine, which spread with extraordinary rapidity, threatened almost all the herds of swine in the land, and through its virulence carried off an extraordinarily large number of animals. Such a devastation among hogs as this epidemic caused has never been experienced before by the younger members of the present generation. An epidemic which has endangered the whole swine-traffic and swine-breeding to such an extent had never been observed in our provinces. At the same time the epidemic showed such polymorphism of phenomena and such deviations in the course of its development that it was difficult for the superficial observer to believe in the etiological unity of all the numerous cases of the disease.

The committee sent out printed lists of questions to the various districts, and it is from these that I make my report; as only seventeen answers have been received, the same must necessarily be somewhat incomplete, and in regard to the clinical observation I have often had recourse to my own experience in the cases which came directly under my charge.

The spread of the disease occurred, according to all reports, mostly through the contact of hog with hog in common pens, during transport, and also in common pastures. It is generally supposed to have been the hogs which were imported from Galicia and Hungary and those bought in Wr.-Newstadt and Biala which communicated the disease to the native Austrian herds. Messrs. Greiner and Weigl have called especial attention to the fact that the manure of sick hogs has often been the means of infecting healthy animals, and there is no doubt that many cases have occurred in pens which were not kept clean. Cases occurred even in pens which were said to have been *disinfected*, and I take occasion to say just here that there is no proceeding which does so much mischief as this disinfection when it is not carried out by men who thoroughly understand their business. No other means has attained such a popularity and none forms such a catch-word in the case of contagious diseases, but none has sunk to such a mere perfunctory performance with very indefinite limits. Among the masses of the people it is common to sprinkle lime-water, green vitriol, carbolic acid, etc., about the places supposed to be infected; but if disinfection is to be effective, it must be carried out with regulations designed to stamp out the disease, particularly by quarantine, as the reports show that a large number of the cases were infected by the manure, or in the pens which were separated from infected ones by very insufficient division-walls and fences.

In one or two cases infection from attendants who visited the infected stalls is supposed to have taken place, showing that too much care cannot be exercised in keeping the hands and particularly the shoes clean in going from pen to pen.

According to the reports and the experience which we have had here, swine of *every breed*, of both sexes, and of every age are equally susceptible of the contagion. The contrary views in the different reports which assert that this or that breed, that this or that sex, show at one time a greater, at another a less, disposition to contagion, give, in the fact that they are so contradictory, the very best proof for the opinion put forth at the beginning of this paragraph. Nevertheless, it is certain that in the case of this epidemic a certain innate immunity is sometimes, though not often, to be observed in this or that individual. Two cases have come under my own immediate notice. Five very well-bred hogs born at the same time (from the same mother) had been kept together in a pen into which the disease had crept from an adjoining one. Three head died, a fourth fell sick and showed the disease in its different stages and remained sickly, but the fifth, although it had been the whole time in the closest contact with the infected hogs, was not sick a single hour, nor did it show later the slightest symptoms of infection. In the second case, a sow with four sucking pigs, succumbed to the disease, while two others remained wholly untouched by the epidemic.

(To be continued.)

